# Clinical Outcomes Following Repair of Acute Patellar Tendon Injuries at a Tertiary Trauma Centre: A 16-Year Retrospective Series

**DOI:** 10.7759/cureus.111133

**Published:** 2026-06-19

**Authors:** Osasenaga Bencharles, Neil Ashwood, Mohammed Khatir, Akhshay George, James Heath

**Affiliations:** 1 Trauma and Orthopaedics, University Hospitals of Derby and Burton NHS Foundation Trust, Burton, GBR; 2 Trauma and Orthopaedics, East Kent Hospitals University NHS Foundation Trust, Kent, GBR; 3 Trauma and Orthopaedics, University Hospitals Coventry and Warwickshire NHS Trust, Coventry, GBR

**Keywords:** anchor suture repair, avulsion fracture, knee extensor mechanism, knee trauma, patella tendon rupture

## Abstract

Background

Patellar tendon rupture is an uncommon but functionally debilitating injury requiring prompt surgical repair to restore the knee extensor mechanism. Evidence regarding the effect of short in-hospital delays to surgery on postoperative outcomes remains limited. This study evaluated short-term clinical outcomes following acute patellar tendon repair at a tertiary trauma centre over a 16-year period.

Methods

A retrospective case series was conducted using a prospectively maintained trauma registry from a tertiary hospital in the East Midlands, United Kingdom (2008-2024). Clinical records, operative notes, and follow-up documentation were reviewed. Data collected included patient demographics, injury characteristics, imaging modality, repair technique, surgeon grade, time to surgery (TTS), postoperative range of motion (ROM), follow-up duration, and complications. The primary outcome was postoperative knee flexion ROM at discharge. Statistical analysis included Pearson correlation and Spearman's correlation for non-parametric analysis, independent-samples t-tests, and one-way ANOVA.

Results

Thirty-four patients underwent operative repair of acute patellar tendon rupture. The cohort had a male-to-female ratio of 7.5:1, with a mean age of 50.7 years. Mean TTS was 2.5 days, and mean postoperative ROM at discharge was 105.4°.

All patients with documented range-of-motion data achieved at least 85° of knee flexion, recorded at the final outpatient review. Complications occurred in nine (26.5%) patients, although most were minor and managed non-operatively. There was no significant association between TTS and postoperative ROM at discharge (r = -0.11, p = 0.59), suggesting that short in-hospital delays within the observed acute treatment window were not statistically associated with ROM at discharge. No statistically significant differences in short-term outcomes were observed between consultant-led and supervised registrar-led procedures, although this comparison was exploratory and unadjusted. Additional preoperative imaging was not associated with a statistically significant increase in TTS.

Conclusion

Primary repair of acute patellar tendon rupture produced satisfactory short-term functional outcomes with a low rate of major complications. Within the observed acute treatment window (0-9 days), no association was identified between time to surgery and knee flexion ROM at discharge, although the study was underpowered to exclude small-to-moderate effects. Supervised trainee-led surgery was associated with short-term outcomes similar to those of consultant-led procedures, although case allocation was not randomised. These findings are exploratory and confined to short-term outcomes. Larger prospective studies incorporating validated patient-reported outcome measures are required to better define prognostic factors following acute patellar tendon repair.

## Introduction

Patellar tendon rupture is an uncommon but clinically significant injury that disrupts the extensor mechanism of the knee and can result in substantial impairment of ambulation and knee function. It accounts for approximately 5-12% of extensor mechanism injuries and has an estimated incidence of 0.48 per 100 person-years [1-3]. Prompt recognition and operative repair are therefore important to restore active knee extension and reduce long-term functional morbidity.

Patellar tendon rupture is reported more frequently in males, with male-to-female ratios approaching 10:1 in some series [4]. It is classically associated with athletic individuals aged 30-40 years, whereas quadriceps tendon rupture is more commonly described in older patients [5]. However, systemic and local risk factors such as chronic patellar tendinopathy, anabolic steroid use, diabetes mellitus, chronic kidney disease, rheumatoid arthritis, obesity, and other comorbid states may predispose patients to tendon degeneration and rupture [6,7].

The typical mechanism involves eccentric contraction of the quadriceps muscle during jumping, landing, running down stairs, or other sporting activities. Ruptures most commonly occur at the inferior pole of the patella, although mid-substance and distal tibial tubercle avulsion injuries are also recognised [6]. Diagnosis is primarily clinical and is based on the combination of a palpable infrapatellar gap, difficulty performing a straight-leg raise, and radiographic evidence of patella alta. The Insall-Salvati ratio remains a simple and inexpensive radiographic aid, with a value greater than 1.2 suggesting patella alta and supporting the diagnosis [8]. Ultrasound or magnetic resonance imaging may be useful in equivocal cases, although additional imaging should not unnecessarily delay treatment when the clinical diagnosis is clear [9].

The principle of "time is tendon" is relevant to patellar tendon rupture. Acute ruptures are generally amenable to primary repair, whereas delayed or chronic presentations may involve tendon retraction, scarring, and poorer tissue quality, often requiring augmentation or reconstruction [10,11]. Common repair options include transosseous tunnel repair, suture anchor fixation, and end-to-end suture repair, with augmentation using cerclage wire or fibre wire loops in selected cases [12,13]. Although outcomes following timely repair are generally favourable, limited evidence exists regarding the effect of short logistical delays within the acute repair window on postoperative function.

The aim of this study was to describe the demographic and clinical characteristics of patients undergoing acute patellar tendon repair in a high-volume tertiary trauma centre and to report short-term postoperative outcomes, defined as knee flexion range of motion at discharge and complications. As a secondary, exploratory objective, we examined whether time to surgery was associated with these outcomes or follow-up duration. Given the retrospective case-series design, the limited sample size, and the narrow range of time-to-surgery values, these association analyses are hypothesis-generating rather than confirmatory, and no causal inference is intended.

## Materials and methods

Study design and setting

This was a single-centre retrospective case series conducted within the Trauma and Orthopaedics department of a tertiary hospital in the East Midlands, United Kingdom. A prospectively maintained trauma registry was reviewed retrospectively over a 16-year period from 2008 to 2024. Where available, registry data were supplemented by review of clinical notes, operative records, and outpatient follow-up documentation.

Inclusion and exclusion criteria

**Figure 1:**
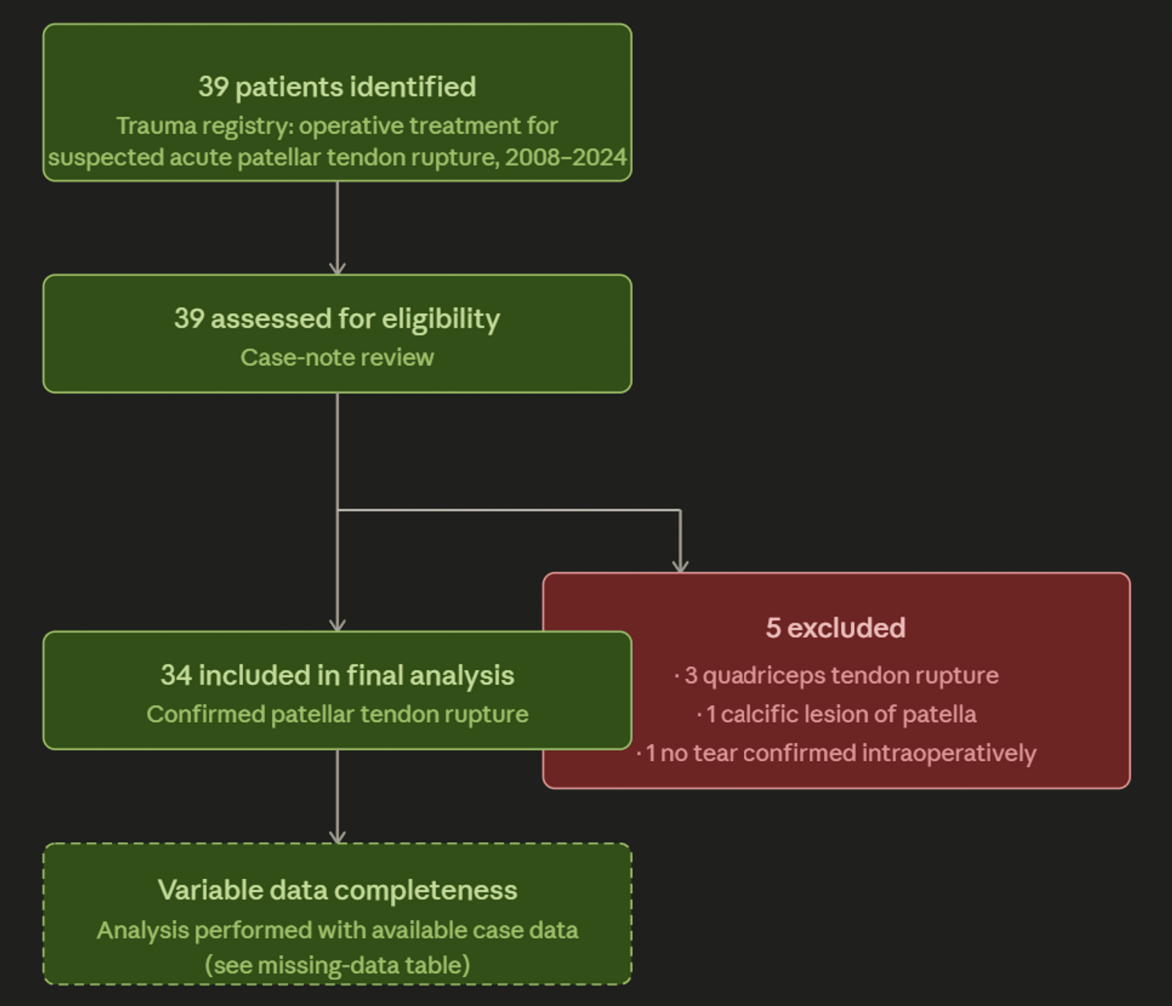
Screening flow diagram Thirty-nine patients were retrieved from the trauma registry, and 34 patients were included for final analysis.

Thirty-nine patients were coded in the trauma registry as having had operative treatment for suspected acute patellar tendon rupture during the study period. Three patients were excluded because they were confirmed on imaging to have quadriceps tendon rupture. One patient was excluded because intraoperative findings did not confirm patellar tendon rupture, and one patient was excluded because the presentation was a calcific lump in the patellar tendon, leaving 34 patients for analysis (see Figure 1). 

Cases with incomplete documentation of individual variables were retained rather than excluded; missing data were recorded and not imputed. Missing data were reported descriptively, and analyses were performed using available cases.

Data collection

Data were extracted on patient demographics, affected limb, mechanism of injury, comorbidities and risk factors for rupture, preoperative imaging modality, anatomical location of tear, repair technique, grade of operating surgeon, time from decision to operate to surgery, postoperative range of motion at discharge, duration of clinical follow-up, and postoperative complications. Missing data were recorded where relevant and were not imputed.

Outcome measures

The primary outcome was knee flexion range of motion (ROM) recorded at the patient's final outpatient review prior to discharge from orthopaedic follow-up, expressed in degrees. This was treated as a pragmatic short-term clinical endpoint rather than a standardised long-term functional outcome, as the timing of discharge varied between patients according to clinical recovery and institutional follow-up practice. ROM was assessed by a clinician using visual estimation and transcribed from the clinical record; assessment was not governed by a dedicated research protocol, and inter-assessor variability cannot be excluded. Discharge from follow-up was a clinical decision made once a functional ROM had been achieved, extensor mechanism function was satisfactory, and the patient was mobilising without brace protection.

Secondary outcomes included length of clinical follow-up, complication rate, and complication type. Follow-up duration was calculated from the date of surgery to discharge from routine outpatient review. A recognised limitation of the registry was non-standardised rehabilitation, missing data, as well as the absence of validated patient-reported outcome measures, such as the Lysholm score, Kujala score, or International Knee Documentation Committee score.

Statistical analysis

Data were analysed using Microsoft Excel (Microsoft Corporation, Redmond, Washington). Continuous variables were reported using mean, standard deviation, and range, while categorical variables were summarised using frequencies and percentages.

Associations between time to surgery and both ROM at discharge and follow-up duration were assessed using Pearson's correlation, with Spearman's rank correlation reported as a non-parametric sensitivity analysis given the small sample and the restricted range of time-to-surgery values.

Independent-samples t-tests were used to compare outcomes between consultant-led and registrar-led procedures. One-way analysis of variance was used to compare time to surgery across imaging modality groups. Statistical significance was set at p < 0.05. Statistical findings were interpreted cautiously and considered exploratory. No formal a priori sample-size calculation was performed, owing to the rarity of the condition and the retrospective design. The study was underpowered to detect small-to-moderate associations; accordingly, non-significant results are interpreted as an absence of evidence of an association rather than evidence of no effect.

Postoperative rehabilitation

Postoperatively, all patients followed the institutional extensor mechanism repair rehabilitation protocol. The operated limb was immobilised in full extension using a hinged knee brace, with progression of weight-bearing and range of motion individualised according to the surgeon's preference and physiotherapy evaluation. For the first two weeks, patients were permitted to weight-bear as tolerated with crutch support, with the brace locked in full extension. From weeks two to eight, knee flexion was advanced incrementally within surgeon-defined limits, typically 0-45° initially, progressing to 0-90° by six weeks, with brace protection maintained during ambulation. At approximately eight weeks, the brace was unlocked and gradually weaned as patients progressed towards full weight-bearing. Routine follow-up was discontinued upon achievement of a functional range of motion, satisfactory extensor mechanism function, and adequate clinical recovery.

## Results

Demographics and baseline characteristics

A total of 34 patients were included in the final analysis after exclusion of three patients who were confirmed on imaging to have quadriceps tendon rupture. One patient was excluded because intraoperative findings did not confirm patellar tendon rupture, and one patient was excluded because the presentation was a calcific lump in the patellar tendon, as shown in Figure 1.

The cohort was predominantly male, comprising 30 (88.2%) men and four (11.8%) women, with a male-to-female ratio of 7.5:1. The mean age at injury was 50.7 years (SD 16.5; range 19-78 years).

The left lower limb was more commonly affected than the right, with 20 (58.8%) and 14 (41.2%) cases, respectively. Age distribution was relatively balanced across groups: 10 (29.4%) patients were younger than 40 years, 11 (32.4%) were aged 40-59 years, and 13 (38.2%) were aged 60 years or older.

Commonly identified systemic risk factors and comorbidities included type 2 diabetes mellitus, chronic kidney disease, gout, hypertension, atrial fibrillation, anticoagulant use, chronic patellar tendinopathy, anabolic steroid use, and obesity. Full demographic data, associated risk factors, and tear location by age group are summarised in Table 1. 

**Table 1:** Association of age group and anatomical location of tear with risk factors The total cohort included 34 patients. Percentages are calculated using the total cohort as the denominator unless otherwise specified. T2DM: type 2 diabetes mellitus; CKD: chronic kidney disease.

Age Group	Mid-substance	Proximal Avulsion	Distal Avulsion	Retinacular Tear	Unknown	Total, n (%)	Risk Factors Present
<40	3	4	0	1	2	10 (29.4%)	Gout, tendinitis, obesity, and anabolic steroids
40–59	3	3	0	0	5	11 (32.4%)	T2DM
>60	2	3	2	1	5	13 (38.2%)	T2DM, CKD
Total	8 (23.5%)	10 (29.4%)	2 (5.9%)	2 (5.9%)	12 (35.3%)	34 (100.0%)	-

Operative records did not document tear morphology in 12 (35.3%) cases, limiting complete anatomical subgroup analysis. Missing data across other key variables included mechanism of injury in 12 (35.3%) cases, postoperative range of motion in seven (20.6%), follow-up data in one (2.9%), and operative technique in 12 (35.3%), as shown in Table 2. 

**Table 2:** Summary of missing data The total cohort comprised 34 patients, which is the denominator for all percentages. Only variables with incomplete documentation are shown; the remaining variables (age, sex, affected side, preoperative imaging modality, time to surgery, operating surgeon grade and complications) were documented in all 34 (100%) patients. Missing data were recorded and were not imputed. ROM: range of motion (knee flexion at discharge).

Variable	Documented, n (%)	Missing Data, n (%)
Tear morphology	22 (64.7%)	12 (35.3%)
Mechanism of injury	22 (64.7%)	12 (35.3%)
ROM at discharge	27 (79.4%)	7 (20.6%)
Follow-up duration	33 (97.1%)	1 (2.9%)
Operative technique	22 (64.7%)	12 (35.3%)

Mechanism of injury

The mechanism of injury was documented in 22 (64.7%) patients. The most common documented mechanism was a fall directly onto the knee in nine (26.5%) patients, followed by twisting injury in six (17.6%), descent of stairs in three (8.8%), hyperextension injury in three (8.8%), and direct laceration in one (2.9%). No mechanism was recorded in the remaining 12 (35.3%) cases, reflecting the limitations inherent to retrospective registry data collection.

Preoperative imaging and time to surgery

Plain radiography alone was the primary diagnostic modality in 21 (61.8%) cases. Additional imaging was utilised in 13 patients, including X-ray with ultrasound in seven (20.6%), X-ray with MRI in three (8.8%), X-ray with CT in two (5.9%), and combined X-ray, MRI, and ultrasound in one (2.9%).

The mean time from decision to operate to surgery (time to surgery, TTS) was 2.5 days (SD 2.0; 95% CI 1.8-3.2; range 0-9 days). Table 3 shows the results of a one-way ANOVA, which demonstrated no statistically significant difference in TTS across the five imaging modality groups (F(4,29) = 0.53, p = 0.71), suggesting that additional imaging was not associated with a statistically significant delay in operative intervention. 

**Table 3:** Relationship between imaging modality and time to surgery (TTS) The total cohort included 34 patients. Imaging-modality values are presented as n (%), with percentages calculated using the total cohort as the denominator. TTS = time from decision to operate to surgical intervention, measured in days. Mean TTS values are calculated within each imaging-modality subgroup and are presented as mean (SD). SD was not calculable for the X-ray + MRI + USS group because this subgroup contained one patient. One-way ANOVA compared mean TTS across imaging-modality groups, F(4,29) = 0.53, p = 0.71. Given the small and unequal subgroup sizes, this result should be interpreted cautiously and is presented descriptively. SD: standard deviation; USS: ultrasound scan; MRI: magnetic resonance imaging; CT: computed tomography.

Imaging Modality	Patients, n (%)	Mean TTS Days (SD)	F-statistic	p-value
X-ray only	21 (61.8%)	2.1 (1.6)		
X-ray + USS	7 (20.6%)	2.6 (1.9)		
X-ray + MRI	3 (8.8%)	2.3 (1.5)		
X-ray + MRI + USS	1 (2.9%)	3.0 (-)		
X-ray + CT	2 (5.9%)	3.0 (0.0)		
Overall (ANOVA)	34 (100.0%)	2.5 (2.0)	F(4,29) = 0.53	p = 0.71

Anatomical tear location and operative technique

Among the 22 (64.7%) cases with documented tear location, proximal avulsion from the inferior pole of the patella was the most common injury pattern in 10 (45.5%), followed by mid-substance tears in eight (36.4%). Distal tibial tubercle avulsions and isolated retinacular tears each accounted for two (9.1%) cases. Transosseous tunnel repair in seven (20.6%) and end-to-end suture repair in 10 (29.4%) were the most frequently employed surgical techniques, reflecting the predominance of proximal avulsion and mid-substance injuries. Suture anchor fixation was used in five (14.7%) cases, primarily in avulsion-type injuries, and operative technique was undocumented in 12 (35.3%) cases.

Postoperative range of motion and follow-up

Postoperative knee flexion data at discharge were available for 27 (79.4%) patients. Mean knee flexion at discharge was 105.4° (SD 15.7°; 95% CI 99.2-111.6°; range 85-135°). All patients with documented range-of-motion data achieved at least 85° flexion, while most achieved 90° or greater, a threshold generally considered functional for activities of daily living.

Short-term functional outcomes stratified by age group are presented in Table 4. One-way ANOVA demonstrated no statistically significant difference in postoperative ROM at discharge between age groups (F(2,24) = 0.37, p = 0.70). The 95% confidence intervals for mean ROM were 87.1°-115.7° in patients younger than 40 years, 94.8°-115.2° in those aged 40-59 years, and 95.9°-120.5° in patients aged 60 years or older. 

**Table 4:** Short-term functional outcomes by age group The total cohort included 34 patients. Age-group sample sizes were: <40 years: 10 patients; 40–59 years: 11 patients; and >60 years: 13 patients. Continuous outcomes are presented as mean (SD), with the number of patients contributing data shown alongside each mean where data were missing. ROM was documented in 27 (79.4%) patients, and follow-up duration was documented in 33 (97.1%) patients. Complications are presented as n (%), with percentages calculated using the age-group sample sizes as the denominator. One-way ANOVA compared mean ROM across age groups, F(2,24) = 0.37, p = 0.70. ROM: range of motion, defined as knee flexion at discharge and measured in degrees; F/U: follow-up duration in weeks; SD: standard deviation.

Age Group	Sample Size, N	Mean ROM° (SD),n	Mean F/U in Weeks (SD), n	Complications, n (%)	ROM ANOVA
<40 years	10	101.4 (15.5), 7 patients	13.9 (9.0), 9 patients	3 (30.0%)	-
40–59 years	11	105.0 (13.2), 9 patients	17.6 (11.5), 11 patients	4 (36.4%)	-
>60 years	13	108.2 (18.3), 11 patients	14.3 (7.8), 13 patients	2 (15.4%)	-
Overall	34	105.4 (15.7), 27 patients	15.7 (9.6), 33 patients	9 (26.5%)	One-way ANOVA: F(2,24) = 0.37, p = 0.70

The mean duration of clinical follow-up was 15.7 weeks (SD 9.6; range 6-48 weeks). Patients were typically discharged once functional ROM had been achieved, and mobilisation without a brace was possible.

Relationship between time to surgery and clinical outcomes

Correlation analysis was performed using available case data. ROM at discharge was documented in 27 (79.4%) cases, while follow-up duration was documented in 33 (97.1%) cases. There was a weak negative correlation between TTS and ROM at discharge using Pearson correlation, which was not statistically significant (r = -0.11, 95% CI: -0.47 to 0.28, p = 0.59). Spearman's rank correlation produced a similar finding, with no statistically significant association between TTS and ROM at discharge (ρ = -0.06, 95% CI −0.43 to 0.32, p = 0.754), consistent across both correlation methods.

Pearson correlation demonstrated a moderate positive association between TTS and follow-up duration (r = 0.35, 95% CI: 0.01 to 0.62, p = 0.045). However, this association was not statistically significant on Spearman sensitivity analysis (ρ = 0.27, 95% CI −0.08 to 0.57, p = 0.124). This finding suggests that the association was weak and non-robust and should therefore be interpreted cautiously. It may reflect the influence of the small sample size, tied TTS values, institutional follow-up practice, or a non-linear data structure. These results are shown in Table 5. 

**Table 5:** Relationship between time to surgery (TTS) and short-term postoperative outcomes The total cohort included 34 patients. Correlations were performed using Spearman’s ρ and  Pearson’s r. The denominators vary across analyses because of missing outcome data: ROM was documented in 27 (79.4%) patients, and follow-up duration in 33 (97.1%) patients. Results are interpreted cautiously, given the small sample size and potential residual confounding by institutional follow-up practices. TTS: time from decision to operate to surgical intervention, measured in days; ROM: range of motion, defined as knee flexion at discharge and measured in degrees; F/U: follow-up duration in weeks; CI: confidence interval.

Outcome	n	Pearson’s r	95% CI	Pearson’s p-value	Spearman's ρ	95% CI	Spearman's p-value
TTS vs ROM at discharge	27	-0.11	–0.47 to 0.28	0.59	-0.06	-0.43 to 0.32	0.75
TTS vs follow-up duration	33	0.35	0.01 to 0.62	0.045	0.27	-0.08 to 0.57	0.12

Surgeon grade and outcomes

Allocation of cases between consultant- and registrar-led lists was determined by theatre list availability rather than by perceived case complexity. Consultant surgeons performed 12 (35.3%) procedures, while registrars performed 22 (64.7%) procedures.

Mean postoperative ROM was 106.8° (SD 16.3°) in consultant-led cases compared with 104.4° (SD 15.7°) in registrar-led cases, representing a mean difference of 2.4° (95% CI: -10.4° to 15.3°). Mean follow-up duration was 14.9 weeks (SD 7.5 weeks) for consultant-led procedures and 16.1 weeks (SD 10.7 weeks) for registrar-led procedures, corresponding to a mean difference of -1.2 weeks (95% CI: -8.4 to 5.9 weeks).

Independent-samples t-tests demonstrated no statistically significant differences in postoperative ROM (t(25) = 0.39, p = 0.70) or follow-up duration (t(31) = -0.35, p = 0.73) between surgeon grades. These results are summarised in Table 6. 

**Table 6:** Comparison of outcomes by operating surgeon grade The total cohort included 34 patients: 12 consultant-led cases and 22 registrar-led cases in weeks. Complications are presented as n (%), with percentages calculated within each surgeon-grade group. Independent-samples t-tests compared the mean ROM and follow-up duration between consultant-led and registrar-led cases. Fisher’s exact test was used to compare complication rates due to small expected cell counts. All cases were performed by registrars under direct consultant supervision; surgeon grade reflects the primary operator. Continuous outcomes are presented as mean (SD), with the number of patients contributing data shown alongside each mean where data were missing. SD: standard deviation; CI: confidence interval; ROM: range of motion, defined as knee flexion at discharge and measured in degrees; F/U: follow-up durationator.

Parameter	Consultant-Led (12 Cases)	Registrar-Led (22 Cases)	Mean Difference (95% CI)	Test Statistic	p-value
Mean ROM° (SD), {n contributing}	106.8 (16.3), {11}	104.4 (15.7), {16}	2.4 (–10.4 to 15.3)	t(25) = 0.39	0.70
Mean F/U weeks (SD), {n contributing}	14.9 (7.5), {12}	16.1 (10.7), {21}	–1.2 (–8.4 to 5.9)	t(31) = –0.35	0.73
Complications, n (%)	2 (16.7%)	7(31.8%)	-	Fisher's exact test	0.43

Complications

Complications occurred in nine (26.5%) patients, most of which were minor and managed conservatively without further surgery. The most common complication was mild extension lag in five (14.7%) patients, typically ranging between 5° and 10°, which generally improved with progressive quadriceps rehabilitation.

One (2.9%) patient developed postoperative stiffness requiring prolonged physiotherapy and manipulation under anaesthesia. One (2.9%) case of wound infection required surgical debridement and split-thickness skin grafting. One (2.9%) case of re-rupture occurred three months postoperatively in a 33-year-old man with documented anabolic steroid use who was non-compliant with postoperative bracing and weight-bearing restrictions; this was subsequently managed with formal reconstruction. One (2.9%) patient demonstrated asymptomatic cerclage wire breakage at routine follow-up, which required no further intervention. No patient reported persistent pain at final clinical review (see Table 7). 

**Table 7:** summary of postoperative complications Overall, complications occurred in 9 of 34 patients (26.5%); each row represents 1 complication in 1 patient. Extension lag (typically 5-10°) improved with quadriceps rehabilitation. Timing is precise only for the re-rupture (3 months, reconstructed at 7 months); other complications were identified at routine outpatient review. Time to surgery did not differ between patients with and without complications (2.78 vs 2.36 days; p = 0.61), and all events occurred within the 0–6-day range. TTS: time to surgery; MUA: manipulation under anaesthesia; T2DM: type 2 diabetes mellitus, AF: atrial fibrillation, STSG: split thickness skin graft.

Age	Sex	Risk Factor/PMhx	Tear Type	Repair Type	TTS	Complication	Timing	Treatment	Outcome
33	M	Anabolic steroid use, non-compliance	Mid-substance tear	End-to-end repair	4	Re-rupture	3 months post-op	Reconstruction	Managed surgically
25	M	Obesity	Proximal avulsion tear	undocumented	4	Extension lag	At follow-up	Physiotherapy	Improved
56	M	No documented risk factors	Not documented	Trans-osseous	1	Extension lag	At follow-up	Physiotherapy	Improved
66	M	Gout, hypertension	Not documented	Trans-osseous	0	Extension lag	At follow-up	Physiotherapy	Improved
72	M	Hypertension, cardiac stents, hypothyroidism	Mid-substance tear	End-to end suture	6	Extension lag	At follow-up	Physiotherapy	Improved
78	M	T2DM, AF on warfarin	Distal avulsion tear	Suture anchor	5	Extension lag	At follow-up	Physiotherapy	Improved
19	F	No documented risk factors	Retinacular tear	End-to-end repair	0	Stiffness	At follow-up	Physiotherapy and manipulation under anaesthesia	Improved
44	M	T2DM	Not documented	Suture anchor	2	Wound infection	At follow-up	Debridement + STSG	Resolved
63	M	No documented risk factors	Not documented	Trans-osseous	3	Supplementary cerclage wire breakage	At follow-up	wire removal, no further procedure	Asymptomatic

Mean TTS was 2.78 days (SD 2.1 days) in patients who developed complications, compared with 2.36 days (SD 1.9 days) in those without complications, representing a mean difference of 0.42 days (95% CI: -1.22 to 2.06 days). Independent-samples t-testing demonstrated no statistically significant association between TTS and complication occurrence (t(32) = 0.52, p = 0.61).

## Discussion

Demographics and epidemiology

This series demonstrated the typical epidemiological profile of patellar tendon rupture, with a marked male predominance. It included 30 (88.2%) male patients, with a male-to-female ratio of 7.5:1, consistent with previously reported ratios approaching 10:1 [4,14]. The mean age of 50.7 years was older than that of the classical athletic population commonly described in the literature but aligns with more recent series reporting increasing incidence in older patients with systemic comorbidities [6,7,15]. Notably, a parallel retrospective study from this centre during the same 16-year period reported a 1.4-times higher rate of quadriceps tendon ruptures than patellar tendon ruptures [16], consistent with published epidemiological data suggesting that quadriceps tendon rupture is more prevalent in older populations [5]. Conditions such as diabetes mellitus, chronic kidney disease, obesity, anticoagulant use, and chronic tendinopathy were frequently identified in our cohort and are likely to have contributed to the older mean age at presentation compared with the purely athletic populations described in earlier series [6,7]. This pattern reflects the evolving clinical reality of extensor mechanism injuries, with an increasing burden of low-energy ruptures in patients with systemic comorbidity rather than high-energy injuries in young athletes.

Proximal avulsion injuries were the most commonly documented tear pattern, followed by mid-substance tears, in keeping with previous reports [1]. However, operative documentation of tear morphology was incomplete in a substantial proportion of cases, limiting detailed subgroup analysis.

Timing of surgery and functional outcomes

The mean time to surgery of 2.5 days was well within the accepted acute repair window described in the literature [6,10]. Within the observed acute treatment window of 0-9 days, no statistically significant association was identified between time to surgery and ROM at discharge (Pearson's r = -0.11, 95% CI: -0.47 to 0.28, p = 0.59). The absence of a statistically significant association should not be interpreted as evidence of equivalence, as the study was underpowered to detect small-to-moderate effects and the correlation analyses were based on relatively small sample sizes. The study, therefore, contrasts essentially immediate surgery with surgery performed within approximately one week and cannot address whether more substantial delays influence outcome. These findings are consistent with previous studies demonstrating satisfactory outcomes following repair within the first two to three weeks after injury [1].

A moderate positive correlation was identified between time to surgery and follow-up duration (Pearson's r = 0.35, 95% CI: 0.01 to 0.62, p = 0.045). On non-parametric sensitivity analysis using Spearman's rank correlation, however, there was no statistical significance (ρ = 0.27, 95% CI −0.08 to 0.57, p = 0.12). Longer follow-up in our cohort may reflect prolonged rehabilitation requirements or institutional factors rather than a direct causal effect of delayed surgery. Nevertheless, these findings may provide reassurance that short unavoidable delays within the acute repair window are unlikely to substantially impair short-term postoperative range of motion or increase complications. However, long-term functional outcomes were not assessed in this cohort.

Surgical technique and surgeon grade

Multiple repair techniques were utilised, reflecting the heterogeneity of injury patterns encountered in routine clinical practice. Although suture anchor fixation has been reported to have a lower re-rupture rate [13,17], the current literature has not demonstrated clear superiority of one repair technique over another regarding functional outcomes or complication rates [18], and our cohort was insufficiently powered to permit meaningful comparative analysis between techniques.

There were no significant differences in range of motion (t = 0.39, p = 0.70, 95% CI for mean difference: -10.4° to 15.3°) or follow-up duration (t = -0.35, p = 0.73, 95% CI: -8.4 to 5.9 weeks) between consultant-led and registrar-led procedures. The numerical differences were clinically negligible. Allocation between consultant- and registrar-led procedures was governed by theatre list availability. Nonetheless, allocation was not randomised, the comparison was unadjusted, and the cohort was too small to exclude residual between-group differences. Although this does not constitute definitive evidence of comparable outcomes, the similar outcomes are reassuring and support the safety of supervised trainee involvement in acute patellar tendon repair within a structured orthopaedic training environment.

Complications

Most complications were minor and manageable conservatively. Mild extension lag was the most common postoperative issue and generally improved with rehabilitation and quadriceps strengthening; this is consistent with the existing literature, in which transient extension lag is a well-recognised sequela following patellar tendon repair and typically resolves with progressive quadriceps rehabilitation [11]. Major complications were uncommon and included isolated cases of wound infection, knee stiffness, and re-rupture.

The single re-rupture occurred in a patient with documented non-compliance with bracing and weight-bearing instructions and a history of anabolic steroid use, both of which have been associated with tendon failure and re-rupture in the literature [6,7].

No statistically significant relationship was identified between time to surgery and complication occurrence, further supporting the observation that short logistical delays within the acute repair period may not substantially influence complication rates, although this finding should be interpreted as hypothesis-generating.

Limitations

Several limitations should be considered when interpreting these findings. The retrospective single-centre design introduces risks of selection bias, incomplete data capture, and unmeasured confounding. The relatively small sample size limits statistical power and increases the likelihood of type II error, particularly in subgroup analyses. No formal sample size calculation was performed due to the rarity of the condition and the retrospective study design.

Functional assessment relied primarily on range of motion and complication data, as validated patient-reported outcome measures such as the Lysholm, Kujala, and IKDC scores were not routinely collected within the registry. Furthermore, the follow-up duration was relatively short, and institutional discharge practices may also have influenced follow-up duration independently of recovery. Mean follow-up of 15.7 weeks captures early ROM recovery only and is insufficient to assess return to sport or work, persistent extensor lag, late re-rupture, or patient satisfaction.

The primary endpoint, ROM at discharge, was recorded at a clinically determined rather than fixed postoperative interval; it reflects both recovery and institutional discharge practice and is not a standardised functional outcome. ROM was not measured under a research protocol, so measurement variability cannot be excluded. Although a common extensor mechanism rehabilitation protocol was used, progression of flexion and weight-bearing was individualised by the surgeon and physiotherapist, introducing variability that may directly affect the ROM endpoint. This limits direct comparability between patients and precludes interpretation of ROM at discharge as a definitive long-term functional outcome. Since ROM was captured at variable time points across patients (overall follow-up range 6-48 weeks), the endpoint is best interpreted as a pragmatic clinical measure reflecting both biological recovery and institutional discharge practice rather than a standardised functional outcome assessed at a uniform time point.

Missing data were present for several key variables, including tear morphology, mechanism of injury, operative technique, and ROM at discharge. Although missingness appeared most likely to reflect retrospective documentation variability rather than systematic exclusion, this cannot be confirmed and may have introduced bias, particularly in subgroup analyses. Therefore, the findings should be interpreted as exploratory and hypothesis-generating rather than confirmatory. Surgeon-grade analysis is limited by the fact that residual unmeasured confounding cannot be fully excluded despite list-based allocation.

Future directions

Future multicentre prospective studies using standardised rehabilitation protocols and validated patient-reported outcome measures are required to better define prognostic factors influencing recovery after patellar tendon repair. Larger collaborative datasets would additionally permit more robust comparisons between repair techniques, timing strategies, and complication profiles. To facilitate this, a minimum dataset for patellar tendon rupture registries should be agreed upon prospectively, incorporating time to surgery, anatomical tear classification, validated patient-reported outcome measures at 6 and 12 months, and return-to-activity status and long-term re-rupture rates. Establishment of such a standardised data framework across trauma centres would substantially improve the quality of evidence available to guide clinical practice.

## Conclusions

This retrospective case series demonstrates that acute patellar tendon rupture can generally be managed successfully with timely primary surgical repair, resulting in an acceptable short-term postoperative range of motion and low rates of major complications.

Within the observed acute treatment window (0-9 days), no association was identified between time to surgery and knee flexion ROM at discharge, although a modest positive association with follow-up duration was observed; given the limited power and narrow time-to-surgery range, these exploratory findings cannot establish that short-term delay is without effect.

Short-term outcomes following consultant- and registrar-led repair were similar, supporting supervised surgical training, though allocation was not randomised and the comparison between groups was unadjusted.

Overall, the interpretation of our findings is limited by the retrospective design, small sample size, absence of validated patient-reported outcome measures, and relatively short follow-up duration. Further prospective multicentre studies using standardised rehabilitation pathways and validated functional outcome measures are required to better define prognostic factors and optimise management strategies following acute patellar tendon repair.
